# In Vitro Bioactivity and Cytotoxicity Assessment of Two Root Canal Sealers

**DOI:** 10.3390/ma18153717

**Published:** 2025-08-07

**Authors:** Yicheng Ye, Sepanta Hosseinpour, Juan Wen, Ove A. Peters

**Affiliations:** School of Dentistry, The University of Queensland, Brisbane 4006, Australia; yicheng.ye1@uq.net.au (Y.Y.); juan.wen@uq.edu.au (J.W.); o.peters@uq.edu.au (O.A.P.)

**Keywords:** bioceramic sealer, osteogenesis, cementogenesis, biocompatibility, periodontal ligament stem cells, PDL cells

## Abstract

The development of bioactive materials in endodontics has advanced tissue regeneration by enhancing the biological responses of periradicular tissues. Recently, calcium silicate-based sealers have gained attention for their superior biological properties, including biocompatibility, osteoconductivity, and cementogenic potential. This study aimed to evaluate the cytotoxicity, biocompatibility, and bioactivity of EndoSequence BC Sealer (ES BC) and AH Plus Bioceramic Sealer (AHP BC) using human periodontal ligament stromal cells (hPDLSCs). Biocompatibility was assessed using MTT, Live/Dead, and wound healing assays. ES BC and AHP BC demonstrated significantly higher cell viability and proliferation compared to AH Plus used as a control. Gene expression analysis via real-time quantitative PCR demonstrated that ES BC, especially in set form, significantly upregulated osteogenic markers—alkaline phosphatase (2.49 ± 0.10, *p* < 0.01), runt-related transcription factor 2 (2.33 ± 0.13), and collagen type I alpha 1 chain (2.85 ± 0.40, *p* < 0.001)—more than cementogenic markers (cementum protein 1, cementum attachment protein, and cementum protein 23). This differential response may reflect the fibroblast-dominant nature of hPDLSCs, which contain limited cementoblast-like cells. This study supports the superior biocompatibility and regenerative capacity of ES BC and AHP BC compared to AH Plus. While in vitro models provide foundational insights, advanced ex vivo approaches are crucial for translating findings to clinical practice.

## 1. Introduction

Among various dental diseases, pulpitis and apical periodontitis resulting from untreated caries remain prevalent issues worldwide [1]. Root canal treatment (RCT) is widely accepted as a primary approach to managing these conditions. RCT involves the mechanical and chemical debridement of the infected root canal system followed by filling and sealing to prevent bacterial re-entry [2,3]. Over the years, root canal therapy has undergone significant advancements in technique and biomaterials. However, despite its high success rate, potential complications, such as persistent infection or reinfection, remain [4,5,6,7].

Among the materials used in root canal therapy, endodontic sealers play a pivotal role in ensuring long-term success. These materials are essential for filling voids and irregularities within the root canal system, preventing microleakage, and contributing to antimicrobial activity within the root canal space [8,9,10]. Following effective mechanical and chemical disinfection, sealers help establish a tight seal, limiting residual bacterial colonization and minimizing the risk of reinfection [11]. Given their function and direct contact with periapical tissues, the physical and biological characteristics of sealers—such as setting time, flow, radiopacity, cytotoxicity, and biocompatibility—must meet high clinical standards [12].

Biocompatibility and cytotoxicity are two of the most important criteria for evaluating sealer performance [13]. A sealer should not only be minimally toxic to host tissues but also support cellular activity and tissue regeneration, especially in the presence of stem cells derived from dental tissues. Cytotoxicity refers to a material’s potential to harm or inhibit the function of vital cells such as periodontal ligament stromal cells (PDLSCs), while biocompatibility describes the ability of a material to coexist with surrounding biological systems without eliciting harmful effects [13,14,15,16]. The pursuit of improved biocompatibility has led to the development of newer biomaterials, particularly calcium silicate-based bioceramics, which are considered more favorable than traditional epoxy resin- or zinc oxide-based sealers due to their bioactivity and regenerative potential [12].

The use of stem cells in dental research has revolutionized our understanding of how endodontic materials interact with human tissues. Stem cells within the periodontal ligament (PDL) are capable of self-renewal and differentiation into multiple cell lineages, including osteoblasts and cementoblasts [17]. These cells not only maintain tissue homeostasis but also contribute to the regeneration of hard tissues and periodontal fibers. PDLSCs, derived from the periodontal ligament, support tooth anchorage and tissue remodeling. They are known for their multilineage potential and share several phenotypic similarities with bone marrow mesenchymal stem cells [18].

An ideal root canal sealer should satisfy a number of clinical and biological criteria. According to the standards established by Grossman decades ago, an effective sealer must demonstrate properties such as strong adhesion to the canal walls, dimensional stability (no shrinkage upon setting), radiopacity, insolubility in periapical fluids, antimicrobial activity, and, most importantly, biocompatibility [19]. Over time, different types of sealers have been developed based on their primary chemical composition. These include zinc oxide-eugenol, calcium hydroxide, epoxy resin, silicone, glass ionomer, and more recently, bioceramic sealers [8,11,20,21].

Despite advances in formulation, no currently available sealer fully meets all of Grossman’s criteria. For instance, zinc oxide-eugenol sealers are known for their antimicrobial activity but have been shown to cause inflammation and exhibit poor long-term biocompatibility [22,23,24,25,26]. Salicylate- and epoxy resin-based sealers like AH Plus offer good handling properties and adequate sealing ability but may exhibit some cytotoxicity due to their chemical constituents [24,27,28,29,30]. Therefore, the development of newer bioceramic sealers has focused on enhancing biological interactions while preserving key physicochemical traits. These bioceramic sealers, often based on calcium silicate, are claimed to be bioactive and inductive of the formation of hydroxyapatite-like structures when in contact with tissue fluids, thus promoting tissue healing and regeneration.

This study was designed to investigate the biocompatibility and bioactivity of two calcium silicate-based root canal sealers—Endosequence BC Sealer (Brasseler, Savannah, GA, USA) and the AH Plus Bioceramic Sealer (Dentsply Sirona, Charlotte, NC, USA). We aimed to assess the impact of these sealers on cell viability, wound healing capacity, and the expression of osteogenic and cementogenic genes in human PDLSCs at two distinct setting time points. Null hypothesis is that the two calcium-silicate sealers would show no difference from epoxy-resin sealers in cytotoxicity, migration, or gene-expression profiles of hPDLSCs.

## 2. Materials and Methods

### 2.1. Preparation of Sealer Extracts

Three root canal sealers were investigated: Endosequence BC Sealer (ES BC) (Brasseler USA), AH Plus Bioceramic Sealer (AHP BC) (Dentsply Sirona, USA), and AH Plus (AHP) (Dentsply Sirona, Germany) (Table 1).

Fifteen cylindrical molds, each measuring 5 mm in diameter and 2 mm in height, were created from medical-grade rubber using sterile syringe tubes as templates for size. These molds were sterilized by exposure to ultraviolet light for 30 min. Following the manufacturers’ instructions, the sealers were mixed (AHP) or dispensed (ES BC and AHP BC) and allowed to set in the molds. Sealers samples were incubated at 37 °C and 90% humidity to set for either 4 h or 2 days, representing the “fresh” condition—where only the initial setting had occurred—and the “set” condition—where the material had fully hardened. Each sealer disk was then placed individually into separate wells of a 12-well plate and immersed in freshly prepared growth medium at 37 °C for 24 h. Extraction was performed following ISO 10993-5 standards [31], maintaining a surface area to medium volume ratio of 1.25 cm^2^/mL. The initial extracts (1:1 ratio) were prepared according to ISO 10993-5 standards [31] and Gaudin et al., 2020 [32]. Subsequently serial dilutions were then performed to obtain extract concentrations of 1:2, 1:4, 1:8, 1:16, 1:32, and 1:64 (*v*/*v*).

### 2.2. Cell Isolation and Culture Conditions

This study received ethical clearance from the University of Queensland (2021/HE000224). Healthy human PDLSCs were obtained from extracted teeth for orthodontic purpose with a non-enzymatic digestion method as previously described [33]. Briefly, cells were cultured in complete in Dulbecco’s Modified Eagle’s Medium (DMEM) (Life Technologies, Carlsbad, CA, USA) supplemented with 10% fetal bovine serum (Thermofisher Scientific Australia, Scoresby, VIC, Australia) and 1% penicillin and streptomycin at 37 °C with 5% CO_2_ humidified incubator. Cells were passaged using 0.25% trypsin when they reached 80–90% confluence and only cells below passage 9 were used.

### 2.3. Cryopreservation and Thawing of Cells

Cells demonstrating good morphology and viability were cryopreserved to maintain consistency for future experiments. Following trypsinization, cells were resuspended in a cryoprotectant mixture (70% DMEM, 20% FBS, 10% DMSO), aliquoted into cryotubes, and placed in an isopropanol chamber before freezing at −80 °C to ensure gradual cooling.

### 2.4. MTT Assay

Cytotoxic effects were quantitatively assessed using a commercial MTT assay (MTT reagent (3-(4,5-dimethylthiazol-2-yl)-2,5-diphenyltetrazolium bromide), Sigma-Aldrich, St. Louis, MO, USA). Human PDLSCs were seeded at 5 × 10^3^ cells per well in 96-well plates. After cell attachment, the medium was replaced with serial dilutions of sealer extracts (1:1, 1:2, 1:4, 1:8, 1:16, 1:32, and 1:64) from both “fresh” and “set” groups. Each dilution was tested in triplicate. Control groups included untreated cells (positive control) and wells containing only dimethyl sulfoxide (DMSO) as blank (negative control). Cells were incubated for 1, 3, and 7 days. At each time point, medium was discarded and replaced with 100 µL of MTT solution (0.5 mg/mL in medium), followed by 4 h of incubation at 37 °C. The resulting formazan crystals were solubilized with 100 µL DMSO, and absorbance was measured at 565 nm using a microplate reader (TECAN Infinite M200 Pro, Männedorf, Switzerland). Proliferation rate (%) was calculated using the formula:

$$Proliferation\;Rate\;\left(\%\;of\;Negative\;Control\right)=\left(\frac{OD\;of\;experimental\;group\;-\;\;OD\;of\;blank\;}{OD\;of\;positive\;control\;-\;OD\;of\;blank}\right)\;\times \;100$$

### 2.5. Live/Dead Cell Staining

For qualitative cytotoxicity analysis, cells were seeded in 24-well plates at 2 × 10^4^ cells per well and exposed to ES BC or AHP BC extract dilutions (1:1 (undiluted), 1:2, and 1:4) for 1 or 3 days. Due to the high cytotoxicity of AH sealer, it was impractical to perform any further biocompatibility testing with dilutions higher than 1:16. Staining solution containing calcein AM and ethidium homodimer-1 was prepared and applied for 30 min. Live cells fluoresced green, and dead cells fluoresced red under confocal laser scanning microscopy. The experiment was conducted in triplicate and protected from light.

### 2.6. Immunocytochemistry

To assess morphological changes in hPDLSCs after exposure to the extracts of ES BC and AHP BC, immunocytochemical staining was performed. Ten thousand hPDLSCs were seeded on coverslips and cultured at 37 °C for 24 h. Following this, cells were incubated with 1:1 (undiluted), 1:2, and 1:4 dilutions of material extracts, while the control group received only phosphate-buffered saline (PBS). The cells were then fixed with 4% formaldehyde (Merck Millipore, Darmstadt, Germany) for 10 min and washed with 5% bovine serum albumin (Sigma-Aldrich, St. Louis, MO, USA) for 30 min. After treatment, cells were stained with AlexaFluor™594-conjugated phalloidin (Invitrogen, Carlsbad, CA, USA), and nuclei were counterstained with DAPI (4′,6-diamidino-2-phenylindole) (D1306, 4′,6-diamidino-2-phenylindole, Thermo Fisher Scientific, Waltham, MA, USA). Imaging was performed using a confocal laser scanning microscope (Nikon C2+, Nikon, Tokyo, Japan). Each condition was tested in triplicate and independently analyzed in three separate experiments.

### 2.7. Scratch Wound Healing Assay

To assess the impact on cell migration, a scratch assay was performed using hPDLSCs seeded in 24-well plates at 5 × 10^4^ cells/well. Once cells reached 80–90% confluence, A 200 µL sterile polypropylene pipette tip was used to create a linear wound; images were captured with a 20 × phase-contrast objective (NA 0.4). Cells were then treated with AHP BC or ES BC sealer extracts 1:1 (undiluted), 1:2, and 1:4, while control groups received no extracts. Images were captured at 0, 6, 24, 48, and 72 h. Black shadows visible along the wound edges are phase-contrast artifacts caused by light reflection. Images were captured with the microscope’s position-mark function to return to the same field; minor positional drift (≤50 µm) may occur. Wound closure was analyzed using ImageJ software (ImageJ v1.53, National Institutes of Health, Bethesda, MD, USA) and migration rates were calculated. The formula to calculate the cell migration rate (%) that was used in this assay is as follows:

$$Cell\;Migration\;Rate\;\left(\%\right)=\left(\frac{Initial\;Wound\;Area\;-\;Wound\;Area\;at\;Time\;Point\;}{Initial\;Wound\;Area}\right)\;\times \;100$$

### 2.8. RT-qPCR for Gene Expression

To evaluate gene expression changes, 5 × 10^4^ cells/well hPDLSCs were seeded in 6-well plates and cultured in DMEM. Cells were then treated with AHP BC or ES BC sealer undiluted extracts, while control group received no extracts. Total RNA was extracted using TRIzol (TRIzol™ Reagent, Thermo Fisher Scientific, Waltham, MA, USA) and quantified using a Nanodrop spectrophotometer (NanoDrop 2000c, Thermo Fisher Scientific, Waltham, MA, USA). cDNA was synthesized, and real-time qPCR was performed using SYBR Green (PowerUp™ SYBR™ Green Master Mix, Thermo Fisher Scientific, Vilnius, Lithuania) to quantify markers of osteogenic and cementogenic differentiation: *RUNX2*, *ALPL*, *COL1A1*, *CAP*, *CP23*, and *CEMP1*, with *β-actin* as a reference (Table 2). Relative gene expression was calculated using the ΔΔCt method.

### 2.9. Statistical Analysis

All data were analyzed using GraphPad Prism 10 (Version GraphPad, La Jolla, CA, USA). Results were expressed as mean ± standard deviation. Two-way one-way analysis of variance (ANOVA) with Tukey’s post hoc test was used for cytotoxicity and migration comparisons, while one-way ANOVA with Tukey’s test was used for gene expression. A *p*-value < 0.05 was considered statistically significant.

## 3. Results

### 3.1. Cell Proliferation Rates

**Figure 1:**
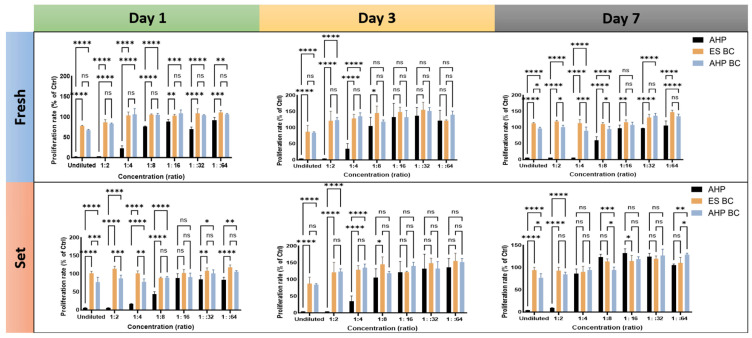
Cell proliferation assessed by MTT assay at various sealer extract dilutions (1:1 (undiluted) to 1:64) after exposure periods of 1 and 3 days. Sealers tested were AH Plus (AHP), EndoSequence Bioceramic Sealer (ES BC), and AH Plus Bioceramic Sealer (AHP BC). Data represent mean ± standard deviation (n = 3). Statistical significance was determined by post hoc Tukey tests; **** *p* < 0.0001, *** *p* < 0.001, ** *p* < 0.01, * *p* < 0.05, ns: non-significant.

The cell proliferation and viability of hPDLSCs cultured with sealer extracts were quantitatively assessed using the MTT assay at 1 and 3 days (Figure 1). Cell proliferation rates were significantly influenced by dilution, incubation time, and sealer setting conditions (fresh vs. set). AHP showed the lowest proliferation rates across all tested dilutions, particularly at concentrations below 1:8, where the average cell viability dropped dramatically to approximately 22.5 ± 4.8%. At undiluted and 1:2 dilutions after 3 days, AHP showed significantly lower average proliferation rates (undiluted: 3.6 ± 0.5%; 1:2 dilution: 14.2 ± 2.9%) compared to ES BC (undiluted: 52.7 ± 4.1%; 1:2 dilution: 67.9 ± 3.6%) and AHP BC (undiluted: 47.3 ± 3.9%; 1:2 dilution: 61.4 ± 3.2%). ES BC and AHP BC extracts both demonstrated significantly higher cell proliferation compared to AHP, with ES BC extracts achieving the highest proliferation rates, notably at intermediate dilutions. For example, ES BC fresh extracts at 1:64 dilution significantly increased cell viability after 3 days (126.8 ± 4.5%, *p* < 0.01) relative to the negative control. These findings confirm that ES BC and AHP BC have lower cytotoxicity than AHP, with proliferation effects clearly dependent on dilution and sealer setting conditions.

Due to the pronounced cytotoxicity of AHP at clinically relevant concentrations (undiluted), it was not feasible to include AHP in further assessments, as our aim was to evaluate conditions closer to clinical applications. Thus, only ES BC and AHP BC were considered for subsequent experiments.

### 3.2. Cell Viability and Morphology

**Figure 2:**
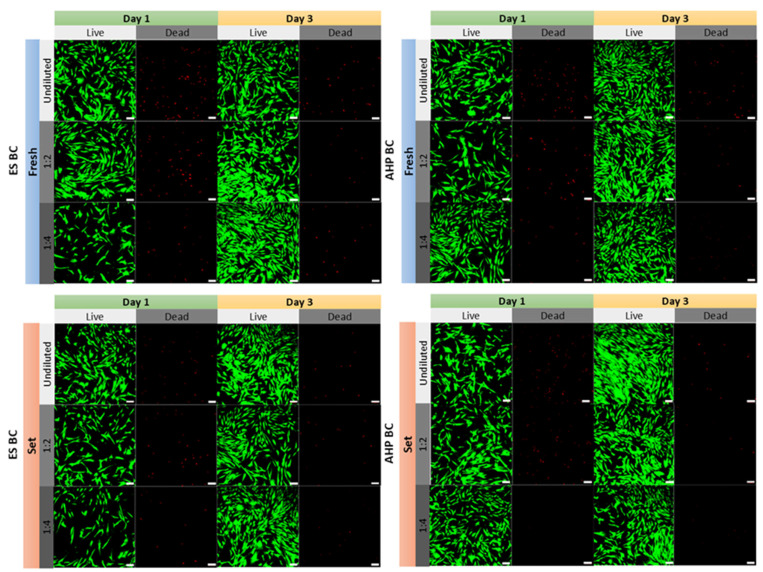
Live/Dead assay illustrating the viability of PDLSCs exposed to extracts of EndoSequence Bioceramic Sealer (ES BC) and AH Plus Bioceramic Sealer (AHP BC), in both fresh and fully set conditions after 1 day and 3 days. Green fluorescence indicates live cells, while red fluorescence indicates dead cells. Scale bars represent 100 µm.

Qualitative assessment of cell viability using the Live/Dead staining assay (Figure 2) confirmed the quantitative MTT results. Cells cultured with extracts from fresh and fully set ES BC and AHP BC sealers demonstrated predominantly live cells (green fluorescence). The highest concentration (1:1 dilution) showed increased numbers of dead cells (red fluorescence), notably for ES BC (fresh) and AHP BC (fresh)groups, consistent with the significant reduction in viability observed in the MTT assay. The viability improved substantially at lower concentrations, with near-total viability (minimal red fluorescence) visible at dilutions of 1:16 and beyond. Cell viability approached control levels at dilutions ≥1:16, although occasional field-to-field variability was observed (Figure 2). The slightly greater red fluorescence seen at the 1:4 dilution of ES BC fresh likely reflects local cell-density differences rather than a reproducible cytotoxic trend.

**Figure 3:**
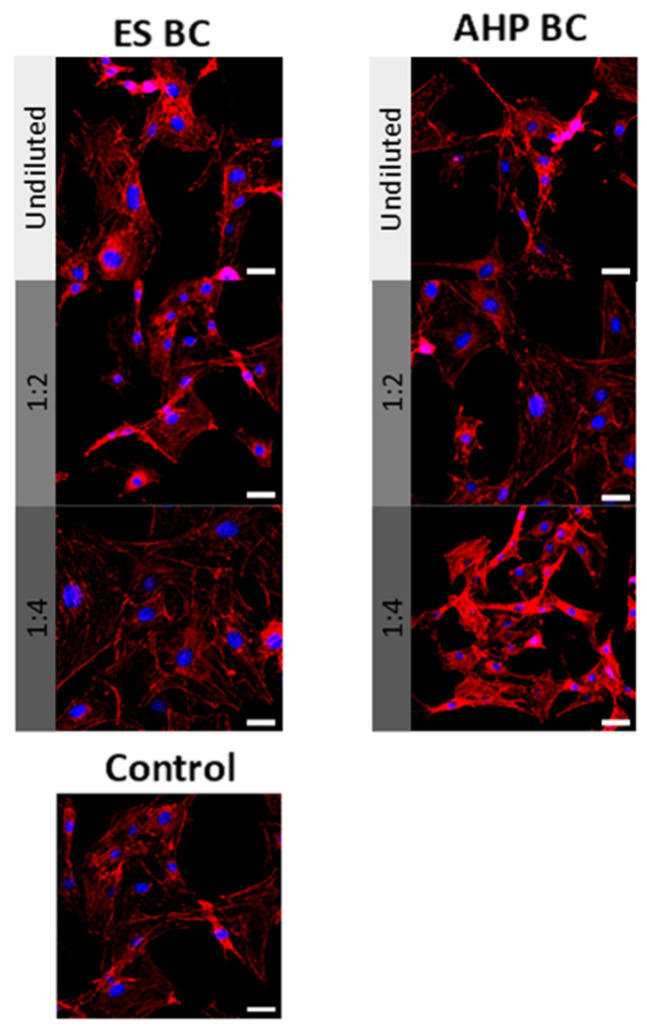
Cytoskeleton staining of human periodontal ligament stem cells (PDLSCs) following 72 h of culture in the presence of fully set EndoSequence Bioceramic Sealer (ES BC) and AH Plus Bioceramic Sealer (AHP BC) extracts compared with the control group. Actin filaments are visualized in red (phalloidin staining) and nuclei in blue (DAPI staining). Scale bars represent 100 µm.

To assess the impact of fully set ES BC and AHP BC extracts on cell morphology and cytoskeletal structure, hPDLSCs were stained with AlexaFluor™594-conjugated phalloidin (red fluorescence) and DAPI (blue fluorescence) after 72 h of exposure (Figure 3). Control cells displayed normal, well-organized, elongated spindle-shaped morphology. Similar intact morphology and robust actin cytoskeletal arrangements were observed in cells treated with fully set ES BC and AHP BC sealers. No observable disruptions or cytoskeletal disorganization were evident, indicating excellent biocompatibility from these fully set sealer extracts at tested concentrations.

### 3.3. Wound Healing and Cell Migration

**Figure 4:**
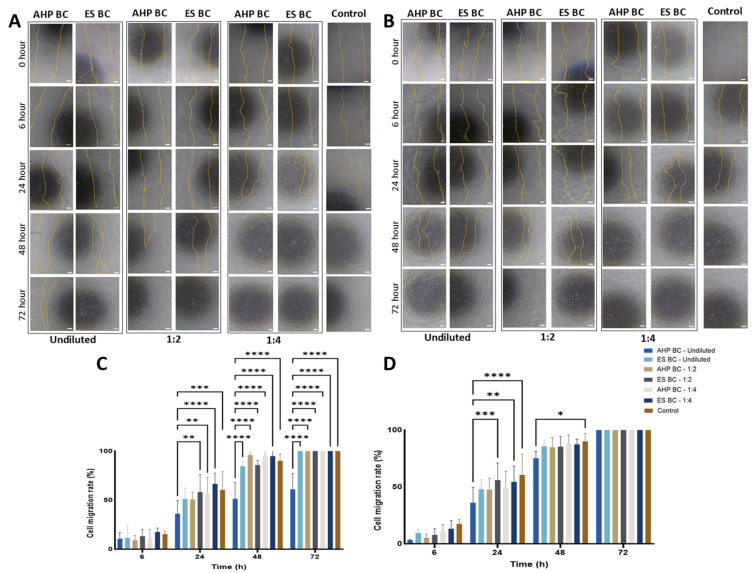
Results from the cell migration (wound healing) assay with human periodontal ligament stem cells (hPDLSCs) exposed to 1:1, 1:2, and 1:4 eluates of EndoSequence Bioceramic Sealer (ES BC) and AH Plus Bioceramic Sealer (AHP BC) after 6, 24, 48, and 72 h. (**A**,**B**) Representative light microscopy images showing the wound closure process, with a scale bar of 200 µm and magnification of ×20. (**C**,**D**) Graphical representation of wound area closure, presented as the percentage of remaining open wound areas relative to the negative control. Panels (**A**,**C**) correspond to fresh sealer samples, whereas panels (**B**,**D**) depict fully set samples. Data represent mean ± standard deviation (n = 3). Statistical significance was determined by post hoc Tukey tests; **** *p* < 0.0001, *** *p* < 0.001, ** *p* < 0.01, * *p* < 0.05.

Cell migration and wound-healing capabilities were evaluated using a scratch assay. Representative images (Figure 4A,B) showed gradual wound closure over 72 h in hPDLSCs cultured with sealer extracts. Quantitative analysis (Figure 4C,D) demonstrated that at 72 h, cells exposed to ES BC (fresh) extracts at 1:4 dilution exhibited significantly faster wound closure (22.3 ± 2.1% remaining open area, *** *p* < 0.0001) compared to the negative control (51.5 ± 3.8%) which is in agreement with the elevated proliferation rates (Figure 1). Similar significant effects were seen with AHP BC (fresh)at the same dilution (28.9 ± 2.5%, **** *p* < 0.0001). Higher concentrations (1:1 and 1:2) showed less effective wound closure, consistent with increased cytotoxicity. For fully set extracts, the ES BC group at 1:4 dilution demonstrated similarly accelerated wound healing (19.7 ± 1.8%, **** *p* < 0.0001), outperforming both the AHP BC (27.4 ± 2.9%, *** *p* < 0.001) and negative control groups, suggesting that these sealer extracts significantly enhance the migration capability of hPDLSCs via stimulating cell proliferations at lower dosage. The accelerated closure reflects enhanced cell migration and reduced cytotoxic stress, not increased proliferation.

### 3.4. Gene Expression of Osteogenic and Cementogenic Markers

**Figure 5:**
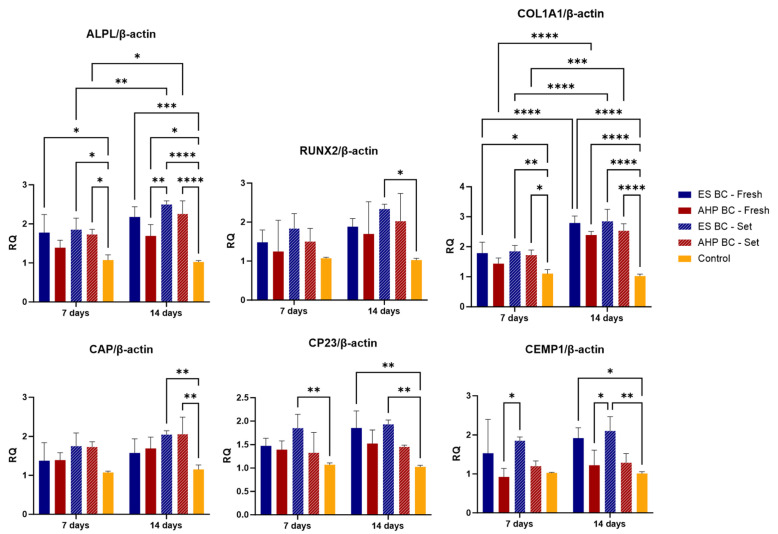
RT-qPCR results showing gene expression of osteogenic and cementogenic markers (*RUNX2*, *ALPL*, *COL1A1*, *CAP*, *CP23*, and *CEMP1*) in human periodontal ligament stem cells (hPDLSCs) cultured with fresh or set EndoSequence Bioceramic Sealer (ES BC) and AH Plus Bioceramic Sealer (AHP BC) undiluted extracts for 7 and 14 days. Gene expression levels are presented relative to the negative and positive control groups. Statistical significance was determined by post hoc Tukey tests; **** *p* < 0.0001, *** *p* < 0.001, ** *p* < 0.01, * *p* < 0.05. Expanded gene names: *RUNX2*: runt-related transcription factor 2, *ALPL*: alkaline phosphatase, *COL1A1*: collagen type I alpha 1 chain, *CAP*: cementum attachment protein, *CP23*: cementum protein 23, *CEMP1*: cementum protein 1.

The expression of osteogenic and cementogenic markers—alkaline phosphatase (*ALPL*), runt-related transcription factor 2 (*RUNX2*), collagen type I alpha 1 chain (*COL1A1*), cementum attachment protein (*CAP*), cementum protein 23 (*CP23*), and cementum protein 1 (*CEMP1*)—were analyzed in hPDLSCs using RT-qPCR at 7 and 14 days following exposure to both fresh and set forms of EndoSequence BC Sealer and AH Plus Bioceramic Sealer (Figure 5). All values were normalized to *β-actin* and presented relative to the control group.

Overall, all experimental groups showed upregulation of osteogenic and cementogenic markers compared to the control, with expression levels generally increasing over time. Among the osteogenic markers, ES BC (set) consistently showed the strongest upregulation at both time points, particularly for *ALPL*, *RUNX2*, and *COL1A1*, all of which reached significantly higher levels than those observed with AH Plus Bioceramic Sealer and the control (*p* < 0.01 to <0.001). AH Plus Bioceramic Sealer showed moderate upregulation of these markers, though not to the same extent. At both 7 and 14 days, ES BC (set) exceeded AHP BC (set) and fresh groups in *ALPL*, *RUNX2* and *COL1A1* expression (*p* < 0.01), whereas some comparisons (e.g., *RUNX2* at day 7) were not significant (*p* > 0.05).

Cementogenic markers were also elevated, though the response was less pronounced than for osteogenic markers. *CAP* and *CP23* expressions were highest in ES BC (set), especially at 14 days, with statistically significant differences compared to AH Plus Bioceramic Sealer and control (*p* < 0.05 to <0.01). *CEMP1* expression followed a similar trend, again with ES BC (set) achieving the greatest expression. These results suggest that EndoSequence BC Sealer promotes both osteogenic and cementogenic differentiation, with a stronger effect observed in osteogenic gene activation.

## 4. Discussion

This study addressed a gap in the literature by directly comparing the biological effects of two calcium silicate-based sealers—EndoSequence BC Sealer and AH Plus Bioceramic Sealer—on human periodontal ligament stromal cells. The use of these cells is highly relevant, as they are among the primary cell populations that interact with root canal sealers in clinical conditions, either directly through apical foramen or indirectly via dentinal tubules and accessory canals. By focusing on this biologically pertinent model, we were able to assess cytotoxicity and differentiation potential in a way that closely reflects the clinical environment.

### 4.1. Cytotoxicity and Biocompatibility

Our results confirmed the severe cytotoxicity of AHP at clinically relevant concentrations, aligning with previous findings [34,35,36]. Both Endosequence BC sealer and AH Plus Bioceramic had considerable biocompatibility, which is in accordance with previous findings [34,37,38,39]. However, the result contradicted a previous study (440), where they compared the cytotoxicity of Endosequence BC sealer and AH Plus and the results suggested that in 24 h both of them showed high cytotoxicity and turned to mild cytotoxicity until six week, which means contributing factors like setting had influenced their outcome [40]. The MTT assay showed that at higher dilutions (1:16 or greater), AHP’s cytotoxicity was mitigated, and cell viability increased, even surpassing 100%. However, it is critical to acknowledge that such high dilutions are unrealistic in clinical settings, where the material is directly applied without dilution. This highlights a major confounding factor in in vitro studies, where the exposure environment does not accurately mimic clinical conditions. Therefore, while dilution experiments offer insight into baseline toxicity, they do not provide translatable clinical implications.

Additionally, the material’s setting condition played a significant role in cytotoxicity. The set extracts of ES BC and AHP BC consistently showed higher cell viability compared to their fresh counterparts. This observation is crucial because, in clinical applications, the sealer sets in situ, potentially in direct contact with living cells within and around the tooth structure. In vitro assessments of fully set materials may not be reflective of clinical reality, where tissue exposure happens progressively as the material sets. This supports the notion that ex vivo 3D culture models might provide more realistic assessments of cytotoxicity and tissue interaction, as suggested in the recent literature [13].

### 4.2. Wound Healing and Cell Migration

Cell migration and wound healing are vital for tissue regeneration following endodontic treatment. Our scratch assay revealed that both ES BC and AHP BC significantly enhanced cell migration compared to AHP. However, neither of the bioceramic sealers demonstrated a statistically significant advantage over the control group, suggesting that while biocompatible, their direct contribution to wound healing might be limited. This contrasts with some previous studies suggesting enhanced migration with these materials [41,42], possibly due to differences in assay methodologies.

### 4.3. Osteogenic and Cementogenic Differentiation

The RT-qPCR analysis demonstrated that ES BC, particularly in its set form, had a stronger stimulatory effect on osteogenic markers (*ALPL*, *RUNX2*, *COL1A1*) than on cementogenic markers (*CAP*, *CP23*, *CEMP1*). This observation is potentially attributed to the nature of the cell population used—hPDLSCs, which primarily consist of fibroblasts with a limited proportion of stem cells or cementoblast-like cells [43,44]. This imbalance may account for the more pronounced osteogenic gene expression compared to cementogenic markers. This is crucial to note as it may reflect the limitations of using monolayer PDL cell cultures to simulate complex tissue interactions.

*ALPL*, *RUNX2*, and *COL1A1* are established indicators of early osteogenic differentiation. Their upregulation, particularly with ES BC, suggests enhanced mineralization potential, a promising attribute for regenerative endodontics [45,46,47,48]. On the other hand, cementogenic markers such as *CAP*, *CP23*, and *CEMP1* are essential for cementum formation and periodontal regeneration [49,50,51]. Although ES BC promoted these markers, the effect was less pronounced compared to osteogenic markers.

The distinct biological properties observed between ES BC, AHP BC, and AHP may be attributed to differences in their chemical compositions. Both ES BC and AHP BC contain tricalcium silicate, but only ES BC includes dicalcium silicate. Importantly, the proportion of tricalcium silicate differs significantly: AHP BC contains only 5–15% tricalcium silicate, whereas ES BC is composed of 20–35% tricalcium silicate and 7–15% dicalcium silicate. This higher content of active calcium silicates in ES BC likely contributes to its enhanced biological performance, as confirmed by Watson et al., who demonstrated that the behavior of calcium silicate cement-based sealers is highly dependent on their composition [52]. Moreover, the stronger gene activation observed with set extracts likely results from a stabilized alkaline pH and sustained calcium-ion release that favor differentiation, whereas fresh extracts may initially impose oxidative or osmotic stress, transiently dampening transcriptional responses. These differences may partly explain why ES BC showed superior osteogenic and cementogenic gene expression compared to AHP BC, particularly in its set condition.

### 4.4. Clinical Implications and Future Directions

While our findings provide foundational evidence regarding the biological properties of ES BC and AHP BC, it is important to acknowledge the limitations of in vitro experiments in predicting clinical outcomes. Two main confounding factors emerged from our study: dilution effects and setting conditions. High dilutions may artificially enhance cell viability, but do not reflect clinical reality where sealers are applied undiluted. Similarly, the biological effects observed in fully set materials may not be representative of the initial phases of tissue interaction. On the other hand, hPDLSCs were selected because, after complete debridement of the root canal system (pulpectomy), they are the primary cells that clinically come into direct or indirect contact with root-canal sealers extruded beyond the apical foramen or diffused through dentinal tubules. Dental-pulp stem cells are absent in this clinical scenario; hence their behavior was not investigated in the present study. Future research should incorporate 3D ex vivo models and real-time assessment during the setting phase to bridge this gap [13].

## 5. Conclusions

In conclusion, this study demonstrated that EndoSequence BC Sealer and AH Plus Bioceramic Sealer exhibit superior biocompatibility compared to AH Plus and enhanced expression of osteogenic and cementogenic markers in hPDLSCs. Notably, ES BC in its set form consistently showed the highest upregulation of key osteogenic markers (*ALPL*, *RUNX2*, *COL1A1*), suggesting its strong potential for promoting mineralization and tissue regeneration. However, the significant variability in cellular responses based on sealer dilution and setting conditions highlights the limitations of in vitro models in predicting clinical outcomes. Nonetheless, this study provides foundational evidence supporting the regenerative potential of ES BC and AHP BC, guiding future research toward optimizing their research application.

## Figures and Tables

**Table 1:** Sealers tested in this study.

Material	Manufacturer	Composition	Lot Number
EndoSequence BC Sealer (ES BC)	Brasseler USA, One Brasseler Blvd, Savannah, GA 31419, USA	Zirconium oxide, tricalcium silicate, dicalcium silicate, calcium hydroxide, thickening agents	23050701
AH Plus Bioceramic Sealer (AHP BC)	Dentsply Sirona, Charlotte, NC, USA	Zirconium dioxide, tricalcium silicate, dimethyl sulfoxide, lithium carbonate, thickening agent	2309000531
AH Plus Jet (AHP)	Dentsply Sirona, Bensheim, Germany	Bisphenol-A epoxy resin, bisphenol-F epoxy resin, calcium tungstate, zirconium oxide, aerosil, iron oxide pigments, amines, silicone oil	2203000322

**Table 2:** Primer sequences for qRT-PCR.

Gene	Forward Primer (5′-3′)	Reverse Primer (5′-3′)	Product Size (bp)	Reference
*RUNX2*	CATCTAATGACACCACCAGGC	GCCTACAAAGGTGGGGTTTGA	~150	Origene (Cat# HP225916)
*ALPL*	TCAGAAGCTCAACACCAACG	GTCAGGGACCTGGGCATT	~120	Qiagen (QuantiTect^®^ Primer Assay, Qiagen, Hilden, Germany)
*COL1A1*	TGACCTCAAGATGTGCCACT	ACCAGACATGCCTCTTGTCC	~140	Qiagen (QuantiTect Primer Assay)
*CAP*	CCTGGCTCACCTTCTACGAC	CCTCAAGCAAGGCAAATGTC	~140	OriGene Technologies Inc., Rockville, MD, USA (Product Code: HP234663)
*CP23*	GGCGATGCTCAACCTCTAACC	GATACCCACCTCTGCCTTGA	~130	OriGene Technologies Inc. (Product Code: HP218292)
*CEMP1*	CCATCCTATCTCTTTGGACCTGG	CCTTGCTTACAGGTGCTGTCCT	~140	OriGene (Cat# HP203762)
*β-actin*	ATTGCCGACAGGATGCAGA	GAGTACTTGCGCTCAGGAGGA	~150	Housekeeping gene

Abbreviations: *RUNX2*: runt-related transcription factor 2, *ALPL*: alkaline phosphatase, *COL1A1*: collagen type I alpha 1 chain, *CAP*: cementum attachment protein, *CP23*: cementum protein 23, *CEMP1*: cementum protein 1.

## Data Availability

The original contributions presented in this study are included in the article. Further inquiries can be directed to the corresponding author.
